# Editorial: Exploring the breast tumor microenvironment: Association to metastasis, novel risk factors and novel treatments and immunotherapies

**DOI:** 10.3389/fonc.2022.1031206

**Published:** 2022-12-07

**Authors:** Karen Elizabeth Nava-Castro, Mariana Segovia-Mendoza, Jorge Morales-Montor

**Affiliations:** ^1^ Laboratorio de Biología y Química Atmosféricas, Instituto de Ciencias de la Atmósfera y Cambio Climático, Universidad Nacional Autónoma de México, Ciudad de Mexico, Mexico; ^2^ Departamento de Farmacología, Facultad de Medicina, Universidad Nacional Autónoma de México, Ciudad de México, Mexico; ^3^ Departamento de Inmunología, Instituto de Investigaciones Biomédicas, Universidad Nacional Autónoma de México, Ciudad de México, Mexico

**Keywords:** breast tumor, tumor microenvironment, metastasis, novel risk factors, novel treatments, immunotherapies

This Research Topic is dedicated to collating the latest information of biological factors associated with the tumor microenvironment and novel anti-cancer therapies The role of the immune system in the eradication of cancers has been investigated for several decades with controversial findings. Researchers studying the tumor microenvironment have uncovered several pathways that up- or downregulate immune cell activity. Research into those pathways has already led to a new class of immuno-oncology treatments known as checkpoint inhibitors, which disrupt immunosuppression and restore T cell activity. There is some controversy in the field, that resulted from a poor understanding of the underlying mechanisms that govern responsiveness. Hence, significant advances have been made concerning regulating the host immune response against cancer, and several immuno-therapeutics have been recently introduced and used clinically. Various studies have examined potential underlying mechanisms involved in resistance and identified various gene products that play pivotal roles in maintaining the resistant phenotype of the cancer cells to cell-mediated immunotherapy. In general, the special issue compiles a series of articles that evaluate different genes and proteins by bioinformatic techniques and novel genomic tools in order to have more effective prognostic tools, but also, once the disease is present, to better understand the resistance mechanisms in the microenvironment.

The scope of this Research Topic will be to provide updated information to scientists and clinicians that is valuable in their quest to gather information, carry out new investigations, and develop novel immuno-sensitizing agents that are both more potent and might be active, whereby the existing ones were not active. This research topic will provide basic and clinical evidence. based on molecular interactions and clinical studies to address the tumor microenvironment, its role on metastasis, novel risk factors and risk and benefits of novel cancer immunotherapy. To present the results of new immunotherapy trials, discussing the state-of-the-art in breast cancer (BC), including targeted therapies approved by the FDA, along with treatments with clinical potential used in basic studies

Wang et al. investigated The Miller– Payne (MP) grading system as a way to evaluate the pathological responses to neoadjunvant chemotherapy (NAC). The Miller-Payne (MP) grading system is currently the most commonly used pathology evaluation system in China, but it estimates only the reduction in primary tumor cellularity after treatment. Miller-Payne system has 5 grades: grade 5 is a pathological complete response in breast; grades 1–4 are partial pathological response according to tumor reduction ratio, from G4 to G1, the degree of tumor reduction gradually decreases. The 70- gene signature was used to classify the prognosis signatures. Their study showed that analysis of MP grades combined with the 70-gene signature with residual NAC-resistant breast samples significantly correlates with disease free survival (DFS). Another article that postulated genetic tools as predictors in BC was Tumor-Derived Exosomal Non-Coding RNAs by Yi et al. Exosomes are key mediators of several processes in cancer that mediate tumor progression and metastasis. These nano-vesicles, when secreted from cancer cells, are enriched in non-coding RNAs (e.g. microRNAs) complexed with the RNA-Induced Silencing Complex (RISC), that mediate an efficient and rapid silencing of mRNAs at the recipient cell, reprogramming their transcriptome. MicroRNAs in circulation encapsulated in exosomes are protected from degradation by a lipid bilayer and might serve as potential non-invasive diagnostic and screening tools to detect early stage cancer, to facilitate treatment options and possible help in curative surgical therapy decisions. The authors try out to elucidate the recent existing research on the functions and mechanisms of tumor-derived exosomal miRNA, lncRNA, circRNA in BC especially in cell proliferation, metastasis, immunoregulation, and drug resistance. In line with that, the special volume also contains other articles involving the identification of N6-Methyladenosine-Related long noncoding RNAs (LncRNAs) for Predicting Overall Survival and Clustering of a Potentially Novel Molecular Subtype of Breast Cancer.N6-methyladenosine modification (m6A) is the most abundant and prevalent RNA modification present in eukaryotic cells and contains three categories of m6A methyltransferases: writer, reader, and eraser. Zhonget al. aimed to identify a signature comprising N6-methyladenosine (m6A)-LncRNAs and molecular subtypes associated with BC. The authors stated that the m6A-LPS and the potentially novel genotype may provide a theoretical basis for further study of the molecular mechanism of BC and may provide novel insights into precision medicine. Furthermore, in the manuscript of Li et al. the authors discovered that based on the co-expression network for bone metastasis of BRCA, they screened key enhancer RNAs (eRNAs) to explore a prognostic model in predicting the bone metastasis by bioinformatics analysis. Enhancers are classically defined as RNA sequences that regulate the gene expression networks underlying distinct cellular identities and cellular responses to environmental cues. Thus, they identified the potential regulatory signaling pathway of SLIT2 in BRCA bone metastasis, which provides a promising therapeutic strategy for the metastasis of this disease On the other hand, Yi et al. identify genes related to the Tumor microenvironment (TME) and prognosis of triple-negative breast cancer (TNBC). A protein-protein interaction (PPI) network was constructed, and a functional enrichment analysis conducted. Their results were verified using Gene Expression Omnibus (GEO) datasets and immunohistochemistry of TNBC patients. CCR5 and CCR2 are structurally related chemokine receptors whose genes share significant sequence homology, probably arising from a gene duplication event. CCR5 is expressed on a broad range of cells, while CCR2 expression is relatively restricted to immune cell types, as monocytes, NK and T lymphocytes, though it can be induced in other cells under inflammatory conditions. They found that CCR2 and CCR5 are key genes in the TME and prognosis of TNBC with the potential of predictive biomarkers in patients with this disease. Furthermore, the Gene Signatures and Cancer Immune Phenotypes Based on m6 were demonstrated by Zhao et al. Authors state that the N6 -methyladenosine (m6) had been considered as a new layer of epitranscriptomic regulation on mRNA processing, stability, and translation. They state that comprehensive evaluation of tumor m6 A modification pattern will enhancing our understanding of the characterization of immune cell infiltration in the tumor microenvironment and promote the responsiveness of BC to immunotherapy. *The Potential Prognostic Role of Oligosaccharide-Binding Fold Containing Protein 2A (OBFC2A) in Triple-Negative Breast Cancer* was approached by Wu et al. Their study aimed to investigate the potential prognostics of TNBC by analyzing BC proteomic and transcriptomic datasets. In addition, biological functional experiments showed that OBFC2A might promote the proliferation and migration of BC cells. The inhibition of OBFC2A expression blocked the cell cycle in the G1 phase and inhibited the transformation from the G1 phase to the S phase. Finally, downregulation of OBFC2A also increased the total apoptosis rate of cells and may be a potential prognostic biomarker for TNBC. Furthermore, Zhu et al. studied the *Value of the Application of Contrast-Enhanced Magnetic Resonance Imaging (CEMRI) Radiomics and Machine Learning in Preoperative Prediction of Sentinel Lymph Node Metastasis in Breast Cancer* to explore the value of machine learning model based on CEMRI radiomic features in preoperative prediction of sentinel lymph node (SLN) metastasis of BC. The authors mentioned that the clinical value of machine learning models based on CE-MRI radiomic features, providing a highly accurate, non-invasive, and convenient method for preoperative prediction of sentinel lymph node metastasis in BCpatients. With the focus on therapy and resistance to treatment Barchiesi et al. studied the Emerging Role of PARP (An enzyme involved in many functions of the cell, including the repair of DNA damage. DNA damage may be caused by normal cell actions, UV light, some anticancer drugs, and radiation used to treat cancer). Inhibitors of PARP-1 are being studied in the treatment of cancer. Also called poly (ADP-ribose) polymerase-1. Inhibitors in Metastatic Triple Negative Breast Cancer. Clinical research areas are investigating PARP inhibitors in combination with other agents. They enrolled patients without germline BRCA mutations: ongoing phase II/III studies and combined PARP inhibitors with immunotherapy. Authors explained that several clinical trials enroll patients with somatic BRCA mutation or patients carrying mutations in genes other than BRCA1/2 involved in the homologous recombination repair pathway. Thus, combining PARP inhibitors with different therapies might overcome the resistance at some point in BC patients. As we mentioned in previous paragraphs, the immune system plays a very important role in stopping tumor growth, but it can also do so in maintaining proliferation. Immune infiltration and specifically lineages of the immune system have been reported to be important in determining response to therapy. Liang et al. delve into the Tumor Mutation Burden (TMB) and the genomic alterations and T cell receptors (TCR) which are Correlated as key predictive indicators of the Efficacy of Neoadjuvant Chemotherapy (NAC) in BC.The authors demonstrated that the TCR index and TMB have significant interaction and may guide neo-adjuvant treatment in operable BC. Response to NAC in tumors with high TCR clonality may be attributable to high infiltration and expansion of tumor specific CD8 positive effector cells. A novel finding in this Special Issue, is that some microRNAs can regulate immune infiltration. Recent discoveries have unveiled thousands of unique non-coding RNAs (ncRNAs) and shifted the perception of them from being junk transcriptional products to yet to be elucidated-and potentially important-RNAs. Most ncRNAs are now known as key regulators in various networks in which they could lead to specific cellular responses and fates. In major cancers, ncRNAs have been identified as both oncogenic drivers and tumor suppressors, indicating a complex regulatory network among these ncRNAs. In that sense, Liu et al. reported that *the non-coding RNAs (ncRNA)-Mediated Overexpression of Ferroptosis Related Gene EMC2 which Correlates with Poor Prognosis and Tumor Immune Infiltration in BC.* The study of Wei et al. evaluated the role of ferroptosis as an iron-dependent programmed cell death process. Although ferroptosis inducers hold promising potential in treating BC, the specific role and mechanism of the ferroptosis-related gene EMC2 in BC have not been entirely determined. Ferroptosis is an intracellular iron-dependent form of cell death that is distinct from apoptosis, necrosis, and autophagy. Extensive studies suggest that ferroptosis plays a pivotal role in tumor suppression, thus providing new opportunities for cancer therapy. The authors mentioned that the EMC2 levels were significantly associated with tumor immune cell infiltration, immune cell biomarkers, and immune checkpoint expression, making to EMC2 gene a new immune therapeutic target in BC. Their study offers a comprehensive understanding of the oncogenic roles of EMC2 across different tumors. In the paper Bulk and Single-Cell Profiling of Breast Tumors Identifies TREM-1 as a Dominant Immune Suppressive Marker Associated With Poor Outcomes, Lance D Miller et al, found out that Triggering receptor expressed on myeloid cells (TREM1) induces the expression of different cytokines. Additionally, TREM1 was discovered by a statistical ranking procedure as top genes for which high expression was associated with reduced response to NAC, but only in the context of immunologically “hot” tumors otherwise associated with a high NAC response rate. Gao et al. in their contribution *3D Extracellular Matrix Regulates the Activity of T Cells and Cancer Associated Fibroblasts in Breast Cancer*, the authors the authors pointed out the relationship between T cell immunosuppression and cancer associated fibroblasts (CAF) induction, which could be of central importance for the BC invasion and may constitute novel therapeutic targets to improve BC outcomes. In their study, Identification of Key Transcription Factors and Immune Infiltration Patterns Associated With Breast Cancer Prognosis Using WGCNA and Cox Regression Analysis, identified three biomarkers related to BC prognosis. Their results provide a framework for the co-expression of transcription factors modules and immune infiltration in BC. In the research, *Spatial Profiling Identifies Prognostic Features of Response to Adjuvant Therapy in Triple Negative Breast Cancer* Kulasinghe et al. delve into the TNBC as an aggressive subtype that has few effective treatment options due to its lack of targetable hormone receptors. The authors says that data provides early insights into the levels of these markers in the TNBC tumor microenvironment, and their association with chemotherapeutic response and patient survival. The authors applied targeted proteomic analysis of both chemotherapy sensitive and resistant TNBC tissue samples. By quantifying 68 targets in the tumor and tumor microenvironment (TME) compartments and performing differential expression analysis between responsive and non-responsive tumors. The authors conclude that increased ER-alpha expression within the stromal compartments is associated with adjuvant chemotherapy response. Similarly, higher expression of fibronectin and lower levels of CD80 were associated with response within tumor compartments. This highlights the importance of studying the tumor microenvironment and not just the tumor as an independent complex. Qiu et al. in their paper *Tumor-Associated Macrophages: Key Players in Triple-Negative Breast Cancer*. TAMs are divided into typically activated M1 subtype and alternately activated M2 subtype, with different expressions of receptors, cytokines, and chemokines. Recent studies demonstrate that TAMs participate in the process of TNBC from occurrence to metastasis and might serve as potential biomarkers for prognosis prediction. In the same line of research, Zhang et al. in the manuscript *Multi-Omics Profiling Suggesting Intratumoral Mast Cells as Predictive Index of Breast Cancer Lung Metastasis*. Zhang discussed that breast cancer lung metastasis has a high mortality rate and lacks effective treatments, for the factors that determine breast cancer lung metastasis are not yet well understood. They used multi-omics data of the TCGA cohort to emphasize the following characteristics that may lead to lung metastasis. Moreover, they found that mast cell fraction can be used as an index for individual lung metastasis status prediction and verified in the 20 human breast cancer samples. The lower mast cell infiltrations correlated with tumors that were more malignant and prone to lung metastasis. This study is the first comprehensive analysis of the molecular and cellular characteristics and mutation profiles of breast cancer lung metastasis, which may be applicable for prognostic prediction and aid in choosing appropriate medical examinations and therapeutic regimens. In addition Chen et al. demonstrated in their manuscript, *Prognostic Significance of Neutrophil-to-Lymphocyte Ratio and C-Reactive Protein/Albumin Ratio in Luminal Breast Cancers With HER2-Negativity* that preoperative evaluation of neutrophil lymphocyte ration (NLR) and C-reactive protein/albumin ratio (CAR) were significant and independent prognostic indicators for luminal BC with HER2-negativity. Nevertheless, the authors highlighted the importance of elevated levels of NLR and poor prognosis in ER+ and HER+ BC. Zhou et al. in the study *Identification of a novel Necroptosis-related Classifier to Predict Prognosis and Guide Immunotherapy in Breast Invasive Carcinoma* pointed out that the role of necroptosis has been little studied within the context of the tumor microenvironment, however, with the help of bioinformatic tools such as genomic variations based on the Gene Expression Omnibus (GEO) and The Cancer Genome Atlas (TCGA) they postulated that various Necroptosis-related genes may govern high sensitivity toward immunotherapy and chemotherapy in invasive BC. Different signaling pathways have been related to proliferation, invasion, resistance to treatment, and metastasis. In this special volume, several authors contributed important research. The paper *Germline Mutational Landscape in Chinese Patients with Advanced Breast Cancer* by Zhang et al. showed that the most prevalent germline mutations in a large cohort of Chinese patients with advanced BC were BRCA1/2 mutations, followed by ATM and RAD50 mutations. Approximately 16.0% (57/356) of patients carry deleterious mutations in the DDR pathway. Patients with breast or ovarian cancer family history were more likely to carry BRCA1/2 mutations, and ones with DDR mutations had worse survival. Their findings suggest that DDR mutations are prevalent in Chinese BC patients who may potentially benefit from treatment with Poly (ADP-ribose) polymerase inhibitors. Moreover, Li et al. in their manuscript *Enhancer RNA SLIT2 Inhibits Bone Metastasis of Breast Cancer Through Regulating P38 MAPK/c-Fos Signaling Pathway*, demonstrated that based on the co-expression network for bone metastasis of BRCA, they screened key eRNAs to explore a prognostic model in predicting the bone metastasis by bioinformatics analysis. Besides, they identified the potential regulatory signaling pathway of SLIT2 in BRCA bone metastasis, which provides a promising therapeutic strategy for the metastasis of BRCA. Li et al. demonstrate that HER2-low tumors could be identified as a more distinct clinical entity from HER2-zero tumors, especially for the HR-positive subgroup. They also established that a more complex molecular landscape of HER2-low breast cancer might exist, and more precise diagnostic algorithms for HER2 testing could be investigated, with the purpose of offering new therapeutic targets for BC treatment. Zhou et al. in their manuscript *Filamin A is a Potential Driver of Breast Cancer Metastasis via Regulation of MMP-1* delve into the fact that there is recurrent metastasis is a major fatal cause of BC. Regretfully, the driving force and the molecular beneath have not been fully illustrated yet. Their study recruited a cohort of breast cancer patients with locoregional metastasis. In summary, this study demonstrates that FLNA may play as a positive regulator in cancer proliferation and recurrence. It provides new insight into BCmetastasis and suggests a potential new therapeutic target for BCtherapy. 20

In the manuscript *Preoperative Pectoralis Muscle Index Predicts Distant Metastasis-Free Survival in Breast Cancer Patients*, Wen- Huang et al. discussed that sarcopenia is related to adverse clinical outcomes in patients with malignancies. Muscle index is a key parameter in evaluating sarcopenia. However, no data is investigating the association between muscle index and distant metastasis in breast cancer. They found that low PMI/T4 is associated with worse DMFS and OS in BC patients. Hao et al. demonstrated that Tumor necrosis factor receptor associated factor 4 (TRAF4) plays an important role in promoting cell proliferation and in inhibiting cell apoptosis induced by Eg5. In summary, their study suggests a new direction for investigating the role of TRAF4 in driving BC progression. Li et al. in the manuscript *Bioinformatics and Experimental Analysis of the Prognostic and Predictive Value of the CHPF Gene on Breast Cancer*, discussed that Chondroitin Polymerizing Factor (CHPF), is an enzyme involved in chondroitin sulfate (CS) elongation and a novel key molecule in the poor prognosis of many cancers. However, its role in the development and progression of BC remains unclear. The authors demonstrated that CHPF transcriptional expression and DNA methylation correlate with immune infiltration and immune markers. Upregulation of CHPF in BC promotes malignant behavior of cancer cells and is associated with poorer survival in breast cancer, possibly through ECM-receptor interactions and the PI3K-AKT pathway. Singh et al. in the work *Circulatory level of Inflammatory cytoskeleton signaling regime proteins in Cancer Invasion and Metastasis* raised new methods to establish a panel of blood-based diagnostic and prognostic biomarkers in metastatic BC. The biomarkers panel are shaped by inflammatory, MAPK and cytoskeletal signaling pathways serum proteins. They found different proteins that were significantly elevated in the serum of BC patients compared to healthy controls. The authors proposed phospho-LIMK, p38α, and phospho-p38α as potent predictive panels of biomarkers for metastatic BC. Datta et al. state that the lack of highly selective ERβ agonists without ERα activity has limited the exploration of ERβ activation as a strategy for ERα+ breast cancer. In this study, the authors demonstrate the efficacy of highly selective ERβ agonists in ERα+ breast cancer cell lines and drug-resistant derivatives, ERβ agonists blocked cell proliferation, migration, and colony formation and induced apoptosis and S and G2/M cell-cycle arrest of ERα+ breast cancer cell lines. Their results demonstrate that highly selective ERβ agonists attenuate the viability of ER+ BC cell lines *in vitro* and suggest that this therapeutic strategy merits further evaluation for ERα+ breast cancer. In the manuscript, *Changes in Pulmonary Microenvironment Aids Lung Metastasis of Breast Cancer* Wu et al. delves into the fact that Breast cancer has become the most common malignant disease in the world according to the International Agency for Research on Cancer (IARC), and the most critical cause of death is distant metastasis. Their review highlights recent findings regarding the alterations of pulmonary microenvironment in lung metastasis of breast cancer, with a focus on various cells and acellular components. On the same line, in their paper *Clinical Relevance of Estrogen Reactivity in the Breast Cancer Microenvironment*, Takeshita et al. demonstrated that BC with high levels of estrogen reactivity had low immune cytolytic activity and low levels of immunostimulatory cells. It also had low levels of stimulatory and inhibitory factors of the cancer immunity cycle. Patients with high estrogen reactivity were also associated with a better prognosis. The authors demonstrated the relationship between estrogen reactivity and the profiles of immune cells and gene expression, as well as survival. The authors highlighted the molecular interaction of the ER with immune actions. The special volume includes works by different authors worldwide, highlighting the importance of using new approaches and novel methodologies that contribute to understanding the mechanisms of progression of breast cancer and its prevention, diagnosis, and treatment. Likewise, it invites researchers to consider the tumor microenvironment as an integral measure for cancer treatment, therapeutic response and clinical outcome.

We hope that our readers will find fascinating and enticing the first-ever Research Topic devoted to *Exploring the Breast Tumor Microenvironment: Association to Metastasis, Novel Risk Factors and Novel Treatments and Immunotherapies* Edited by us.

## Author contributions

All authors listed have made a substantial, direct, and intellectual contribution to the work and approved it for publication.

